# Intraventricular Pneumocephalus as a Complication of Ventriculoperitoneal Shunt

**DOI:** 10.7759/cureus.18392

**Published:** 2021-09-30

**Authors:** Aldo J. F. da Silva, Ana Luisa Malta Doria

**Affiliations:** 1 Pediatric Neurosurgery Division, Santa Mônica Teaching Maternity-Alagoas State University of Health Sciences, Maceió, BRA; 2 Neurosurgery, Faculdade de Medicina Nova Esperança-FAMENE, João Pessoa, BRA

**Keywords:** ventriculoperitoneal shunt, hydrocephalus, pneumocephalus, brain trauma injury, cerebrospinal fluid (csf)

## Abstract

Pneumocephalus is defined as the presence of air in the intracranial cavity, and this complication is rare after ventriculoperitoneal shunt (VPS) surgery. It can be caused by traumatic brain injury (TBI), surgical interventions, and anatomical or spontaneous malformation. We present a case of intraventricular pneumocephalus associated with the placement of a VPS. The patient was a 40-year-old man who had a VPS inserted 10-years ago due to hydrocephalus caused by TBI. He presented to the emergency room with complaints of headache, vomiting, rhinoliquorrhea, and fever. Computed tomography of the skull showed ventricular dilatation with intraventricular pneumocephalus. In a three-dimensional reconstruction, a bone defect was visualized with meningocele at the base of the skull that would explain the cerebrospinal fluid fistula. The meningocele was surgically corrected. After 14 days of antibiotic treatment, a new VPS was placed and the patient progressed satisfactorily. Pneumocephalus associated with VPS is a rare condition that can develop secondary to a combination of the shunt effect and an anatomical defect at the base of the skull. Excessively negative and persistent intracranial pressure of the shunt allows air to enter and fill the existing vacuum through the defect in the skull base. This bone defect may be congenital, due to traumatic brain injury, or a result of hydrocephalus itself. Computed tomography of the skull is an excellent investigation for the visualization of bone defects, and treatment involves a correction of the fistula. Pneumocephalus associated with VPS is rare. The presence of rhinoliquorrhea is a strong indication of the condition. Once the presence of a fistula is confirmed, it should be corrected to prevent worsening of the pneumocephalus.

## Introduction

Pneumocephalus is described as a collection of air or gas in the intracranial cavity and can be located in the epidural, subdural, subarachnoid, intracerebral, or intraventricular space. According to the literature, the subdural space is the most common location. The first case was reported by Lecat in 1741, but the condition was described later by Thomas in 1866 [1]. In 1913, Luckett described the first case of intraventricular pneumocephalus detected on imaging [2]. However, the term pneumocephalus was only coined in 1914 by Wolf. Ventriculography or pneumoventriculography was the method used for locating intracranial tumors in 1920 by Dandy [3]. In 1975, Pitts et al. described the first case of air influx due to a defect at the base of the skull [4]. In the absence of intracranial tumors or infection, pneumocephalus is caused by head trauma, surgical intervention (in particular endonasal sinus surgery), anatomical malformation, or even spontaneously [1]. Clinically, the patient may be asymptomatic with a small pneumocephalus or symptomatic with a large and tension pneumocephalus [2,5].

We report a rare case of intraventricular pneumocephalus that occurred years after the placement of a ventriculoperitoneal shunt (VPS).

## Case presentation

**Figure 1:**
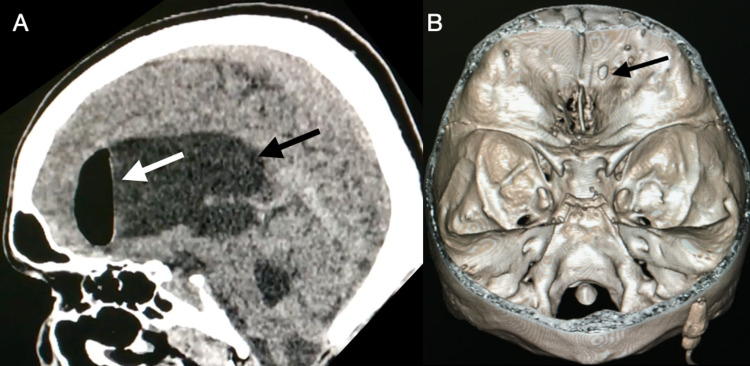
Computed tomography. (A) CT scan of skull sagittal showing intraventricular pneumocephalus (white arrow) and ventricular dilatation (black arrow); (B) three-dimensional computed tomography reconstruction showing left frontobasal bone defect (black arrow). CT: computed tomography.

**Figure 2:**
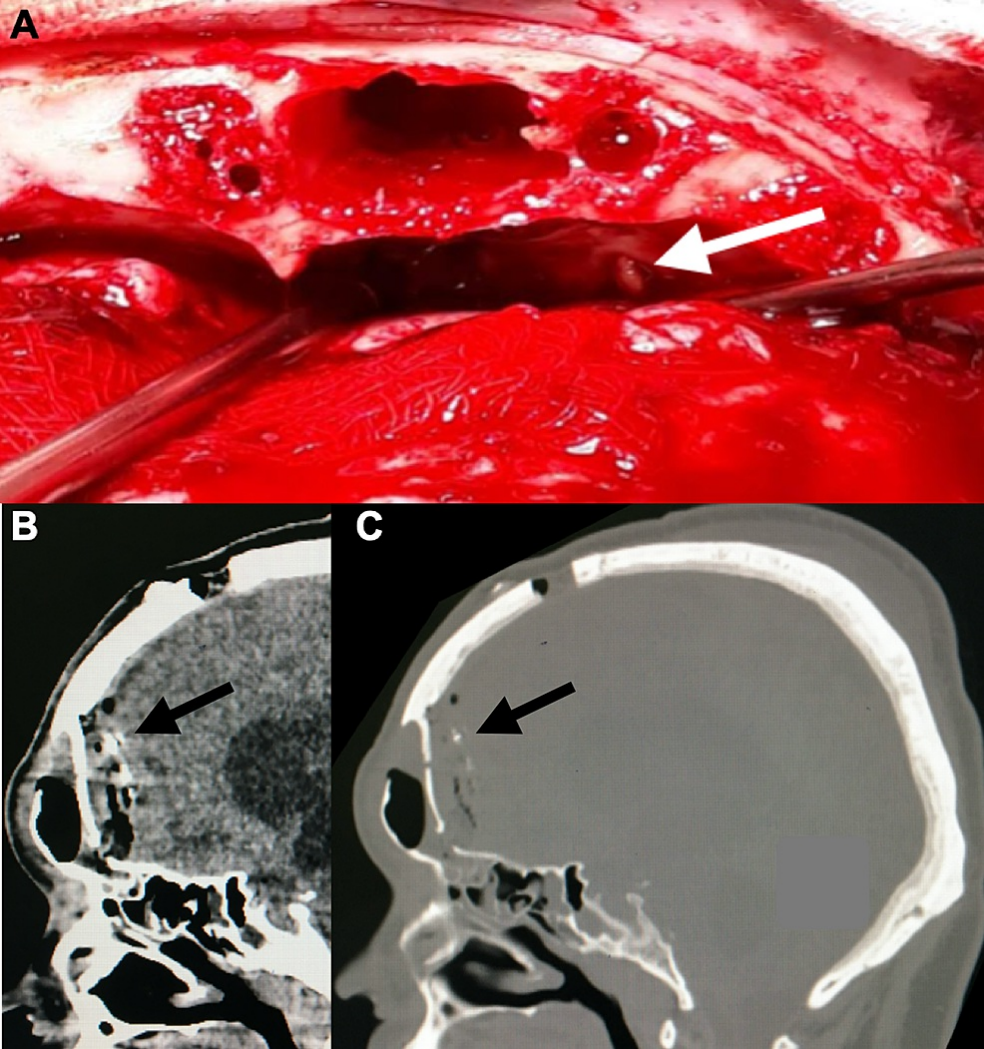
Surgery. (A) Small left frontobasal meningocele (white arrow); (B) CT scan of skull sagittal  showing interposition of the galea aponeurotica in the frontobasal region (black arrow); and (C) bone window showing interposition of the galea aponeurotica in the frontobasal region (black arrow). CT: computed tomography.

The patient was a 40-year-old man with a VPS inserted 10-years ago due to hydrocephalus after a traumatic brain injury. He was admitted to the emergency department with a clinical presentation of headache, vomiting, and fever for 30 days. He also complained of rhinorrhea. Computed tomography (CT) of the skull showed the shunt catheter well positioned in the ventricle, intraventricular pneumocephalus, and ventricular dilatation (Figure 1A). Left frontobasal bone defects were visualized in the three-dimensional (3D) reconstruction (Figure 1B), which could explain the cerebrospinal fluid (CSF) fistula. As there is no endoscope in the institution, it was performed bicoronal craniotomy in which a small left frontobasal meningocele was visualized (Figure 2A). The meningocele was closed with the interposition of the galea aponeurotica in the frontobasal region (Figure 2B, 2C) and performed a frontal sinuses cranialization. Then, the VPS was removed, an external ventricular drain was placed, and ventriculitis (WBC 2100/mm^3^, neutrophils 90%, glucose 20 mg/dl, protein 230 mg/dl, microbiology Streptococcus pneumoniae) was treated with ceftriaxone for 14 days. After this period, with the ventriculitis treated, a new VPS was placed and the patient progressed satisfactorily and was discharged from hospital on the third postoperative day.

## Discussion

Pneumocephalus associated with VPS is a rare condition that can develop secondary to a combination of the shunt effect and an anatomical defect at the base of the skull. Excessively negative and persistent intracranial pressure (ICP) exerted by the shunt allows air to enter and fill the existing vacuum through the defect in the skull base [6].

In the case reported herein, pneumocephalus developed 10-years after insertion of the VPS, and chronic negative intracranial pressure exerted by the shunt led to air leakage through the site of a bone defect. Another possible situation is shunt dysfunction somewhere in the 10-years and increased ICP leading to an encephalocele due to the defect causing fistula, air intake, and pneumocephalus. This bone defect can be congenital or even a consequence of previous trauma to the skull base.

In some situations, the presence of intracranial air may be associated with serosanguineous nasal drainage, which is usually a combination of nasal secretions, blood, mucus, and eventually CSF [7]. Air can access to the intracranial cavity when nasal pressure exceeds ICP in the presence of a space connecting the basal structures to the paranasal sinuses [5]. In such cases, a CSF fistula should be suspected and imaging studies may aid in the diagnosis and allow the observation of a significant amount of intracranial air. High-resolution (HR) CT and magnetic resonance imaging (MRI) cisternography are the methods of choice for the primary investigation of CSF leaks. If these methods fail, there is also CT cisternography, radionuclide cisternography, fluorescence cisternography, and diagnostic nasal endoscopy [8]. In the present case, the defects at the base of the skull that explained the CSF fistula were detected by computed tomography of the head.

The clinical presentation varies depending on the location and mass effect of the pneumocephalus that may increase ICP [9]. The patient may develop a headache, which is the most common symptom, and other neurological symptoms, such as vomiting, blurred vision, syncope, aphasia, motor deficit, and ataxia. In 1983, Jooma and Grant described the sound of sneezing inside the head, “bruit hydroaerique,” as a pathognomic symptom of pneumocephalus [10].

The correct diagnosis is important for treatment, and the latter requires knowledge of the etiology of the communication between the intracranial region and air in the cranial base. Management will vary depending on the clinical presentation, etiology, extent, volume, and progression of the air entrapment [2]. In most patients, the treatment aims at the primary closure of the defect of the base of the skull, ceasing the egress of CSF as well as the influx of air [1,11]. This was the case of the patient described herein, for whom the defect with meningocele at the base of the skull was visualized and corrected.

With regard to the shunt, temporary externalization is sometimes required while the fistula is resolved [1]. Another treatment option is endoscopic repair, which proves to be an effective and safe method even in small defects [8,12].

Moreover, whether patients who are at a higher risk of developing post-shunt pneumocephalus should have a high-pressure valve inserted as a primary measure needs to be established or more likely an adjustable system [13].

## Conclusions

Pneumocephalus associated with VPS is a potential diagnosis when the patient has a headache, vomiting, and, in particular, has rhinorrhea. In the present case, the intraventricular pneumocephalus may have been spontaneous after placement of the VPS or caused by a defect at the base of the skull as a consequence of previous head trauma. Regardless of the etiology, the fistula has to be treated to avoid future complications. The example of this patient who was treated and in the two-month follow-up is doing well.
